# The faster the better? Time to first CT scan after admission in moderate-to-severe traumatic brain injury and its association with mortality

**DOI:** 10.1007/s10143-020-01456-3

**Published:** 2020-12-18

**Authors:** Marius Marc-Daniel Mader, Roman Rotermund, Rolf Lefering, Manfred Westphal, Marc Maegele, Patrick Czorlich, the TraumaRegister DGU

**Affiliations:** 1 Department of Neurosurgery, University Medical Center Hamburg-Eppendorf, Martinistr. 52, 20246 Hamburg, Germany; 2 Institute of Research in Operative Medicine (IFOM), University Witten/Herdecke, Cologne, Germany; 3 Department for Trauma and Orthopedic Surgery, Cologne-Merheim Medical Center, University Witten/Herdecke, Cologne, Germany; 4 Committee on Emergency Medicine, Intensive Care and Trauma Management (Sektion NIS) of the German Trauma Society (DGU), Berlin, Germany

**Keywords:** Traumatic brain injury, Trauma, Register, Computed tomography, Mortality, Admission

## Abstract

Fast acquisition of a first computed tomography (CT) scan after traumatic brain injury (TBI) is recommended. This study is aimed at investigating whether the length of the period preceding initial CT scan influences mortality in patients with leading TBI. A retrospective cohort analysis of patients registered in the TraumaRegister DGU® was conducted including adult patients with TBI, defined as Abbreviated Injury Scale_Head_ ≥ 3 and GCS ≤ 13 who had been treated in level 1 or 2 trauma centers from 2007–2016. Patients were grouped according to time intervals either from trauma or from admission to CT. A total of 6904 patients met the inclusion criteria. Mean time period from trauma to hospital admission was 68.8 min. From admission to first CT, a mean of 19.0 min elapsed. Trauma severity was higher in groups with a longer duration from trauma to CT as represented by a mean (± standard deviation) Injury Severity Score (ISS) of 19.8 ± 9.0, 20.7 ± 9.3, and 21.4 ± 7.5 and similar distribution of mortality of 24.9%, 29.9%, and 36.3% in the ≤ 60-min, 61–120-min, and ≥ 121-min groups, respectively. An adjusted multivariable logistic regression model showed a significant influence of the level of the trauma center (*p* = 0.037) but not for interval from admission to CT (*p* = 0.528). TBI patients with a longer time span from trauma to first CT were more severely injured and demonstrated a worse prognosis, but received a CT scan faster when duration from admission is observed. The duration until the CT scan was obtained showed no significant impact on the mortality.

## Introduction

Despite the efforts taken to improve care and outcome of patients suffering from traumatic brain injury (TBI), the burden of TBI is still associated with large medical and socio-economic problems [15, 19]. A recent publication of the TraumaRegister DGU® (TR-DGU) reported a rate of app. 8220 cases per year with an incidence of 10.1/100,000/year for a moderate or severe TBI in Germany between 2013 and 2017 [15]. To confirm a clinically suspected TBI, it is deemed necessary to carry out an imaging diagnostic, in most cases a computed tomography (CT), which represents the “gold standard” [18]. This fact is reflected by the CENTER-TBI consortium that regards a 24/7 availability of CT scan and radiologist review as a quality indicator in the treatment of TBI patients [10]. Timely access to computed tomography (CT) has been defined as a major prerequisite for the participation of trauma centers within the TraumaNetzwerk DGU® [3]. These recommendations are mainly based on a landmark publication by Huber-Wagner et al. in 2009 demonstrating that the integration of a whole-body CT into early trauma care significantly reduced the mortality [8]. In an Austrian multi-center study, the authors recommend to perform the first CT scan within 20–30-min upon arrival [2]. Duration from admission to CT scan was shown to be associated with the localization of the CT scanner in close relation to the trauma room which had also a beneficial effect on mortality [9]. The REACT-I trial also demonstrated a significant reduction of the duration with the CT localized in the trauma room but without an effect on outcome which is similar to another study [18, 20]. Overall, not only is the data on the duration from admission to the first CT scan in TBI patients sparse but also is the impact of the duration on mortality rarely evaluated in detail [2, 4, 12, 15, 16, 19].

The aim of this retrospective multi-center analysis based on prospectively collected data from the TR-DGU was to investigate the impact of the duration from admission to first cranial computed tomography and its association with in-house mortality.

## Material and methods

### TraumaRegister DGU®

The TR-DGU was founded in 1993. The aim of this multi-center database is a pseudonymized and standardized documentation of severely injured patients. Data are collected prospectively in four consecutive time phases from the site of the accident until discharge from hospital: (A) prehospital phase, (B) emergency room (ER) and initial surgery, (C) intensive care unit (ICU), and (D) discharge. The documentation includes detailed information on demographics, injury pattern, comorbidities, pre- and in-hospital management, ICU course, and relevant laboratory findings including data on transfusion and outcome of each individual. The inclusion criterion is admission to hospital via the emergency room with vital signs and subsequent transfer to the ICU or intermediate care unit or death before admission to the ICU.

The infrastructure for documentation, data management, and data analysis is provided by the AUC (*Academy for Trauma Surgery*), a company affiliated to the *German Trauma Society*. The scientific leadership is provided by the *Committee on Emergency Medicine, Intensive Care and Trauma Management* (*Sektion NIS*) of the German Trauma Society. The participating hospitals submit their pseudonymized data into a central database via a web-based application. Scientific data analysis is approved according to a peer review procedure established by *Sektion NIS*.

The participating hospitals are primarily located in Germany (90%), but a rising number of hospitals of other countries contribute data as well. Currently, approximately 33,000 cases from over 650 hospitals are entered into the database per year. Participation in the TR-DGU is voluntary. For hospitals associated with TraumaNetzwerk DGU®, however, the entry of at least a basic data set is obligatory for reasons of quality assurance. The basic data set is mostly provided by smaller hospitals and contains only a limited range of variables, e.g., no surgical procedures and no times from admission to CT which was analyzed in this study. The standard data set with more detailed information is mostly submitted by high-level trauma centers.

The present study is in line with the publication guidelines of the TraumaRegister DGU® and registered as TR-DGU project ID 2018-004. Furthermore, it was reported to the local ethic committee (WF-059-18).

### Study cohort and variables

**Fig. 1:**
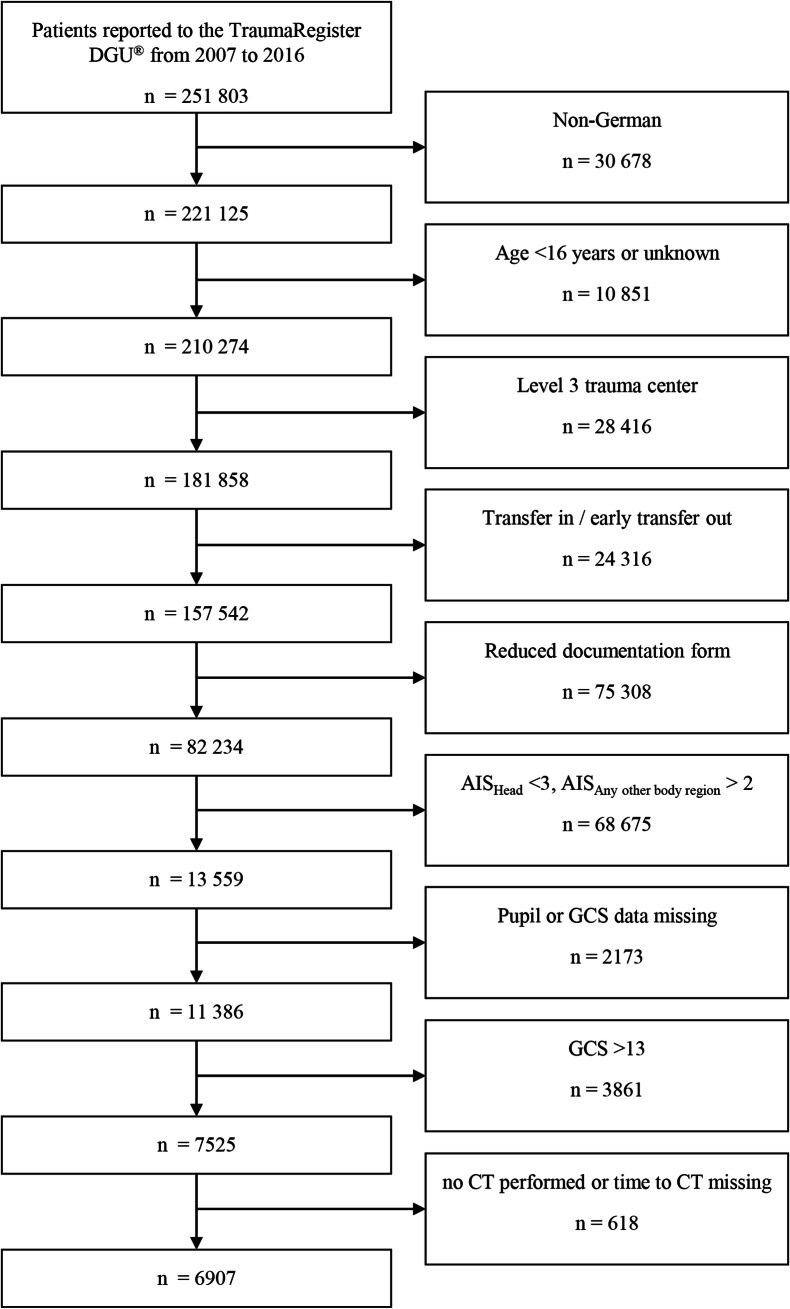
The flowchart describes the exclusion and inclusion criteria. AIS Abbreviated Injury Scale, CT computed tomography, DGU German Trauma Society (Deutsche Gesellschaft für Unfallchirurgie), GCS Glasgow Coma Scale

Although the TR-DGU database comprises a wide variety of information for each case, only patients ≥ 16 years of age treated in participating German level I and II hospitals between 2007 and 2016 with a predominating moderate-to-severe TBI (defined as an Abbreviated Injury Scale (AIS) of the head score ≥ 3 and an AIS in any other body ≤ 2) and a Glasgow Coma Scale (GCS) ≤ 13) were potentially eligible for the analysis. Patients documented only with the basic data set were excluded as the basic data set does not include information on CT times. Patients who were early (< 48 h) transferred to a different hospital were not considered because no outcome information for these patients is available in the TR-DGU database. Missing data of the GCS or pupil status were also an exclusion criterion as well as patients with no CT or a missing time value. The selection process is described in detail in Fig. 1.

Primary outcome parameter in this analysis was the in-house mortality.

Variables extracted from the TR-DGU included basic demographic data, trauma mechanism, and American Society of Anesthesiologists (ASA) physical status classification. Parameters of trauma severity were Injury Severity Score (ISS), AIS of different body regions, and the Revised Injury Severity Classification, version II (RISC-II) predicting the risk of death as discussed by Baker et al. [1, 14]. The RISC-II score has been validated for mortality prediction depending on the clinical status in the emergency room of a large number of patients included into the TR-DGU data set [14]. It considers the AIS severity levels of both worst and second-worst injuries and head injury as well as the variables age, sex, pupil reactivity, and size; preinjury health status; blood pressure; acidosis; coagulation; hemoglobin; and cardiopulmonary resuscitation (CPR). The RISC-II score (higher value means better survival) is transformed into a risk of death estimator using the logistic function.

Additional variables extracted included GCS, rate of whole-body CT, rate of abdominal sonography and chest X-ray in the trauma room, time from trauma to hospital, and total time in the trauma room. For evaluation of time to CT, two variables were calculated: “time from trauma to first CT” included preclinical and trauma room management whereas “time from admission to first CT” only considered the timeframe within the hospital until CT.

### Statistical methods

Statistical analyses were performed using SPSS statistical software (SPSS Version 24.0, IBM Inc., Armonk, New York, USA). Data are presented as mean ± standard deviation (SD) for continuous variables and as numbers and/or percentages for categorical variables respectively as median and interquartile range (IOR). Values of times from trauma to admission, respectively, trauma to first CT and admission to first CT are presented in both mean ± SD and median plus IQR to compare the results with the literature.

In order to assess the impact of the different mentioned time spans on mortality, standardized mortality ratios (SMR) and corresponding 95% confidence intervals (CI) were calculated using the RISC-II score to compare the observed mortality with the expected mortality. Finally, a multivariable logistic regression analysis was performed to assess the independent impact of the trauma center level and time from admission to the CT scan on mortality. The model was adjusted for the RISC-II score. Results were presented as odds ratios (OR) with corresponding 95%CI.

## Results

In accordance with the selection process shown in Fig. 1, 6907 patients met the inclusion criteria. The study cohort showed a male predominance (66.4%) and a mean age of 58.1 (SD 21.7) years. The most frequent trauma mechanism was low fall from less than 3 m. Mean ISS was 20.7. TBI was critical (AIS 5) or maximal (AIS 6) in 42.4% of patients. Anisocoric or bilaterally dilated pupils were present in 32.8% of patients. Mean time period from trauma to hospital admission was 68.8 min. From admission to first CT, a mean of 19.0 min elapsed. This time period was shorter in level 1 trauma centers. Mortality was 31.7% with early mortality within 24 h accounting for almost half of fatalities. A detailed description of the study cohort is provided in Table 1.

**Table 1:** Demographic, clinical, and outcome data of patients

Demographics and clinical data	Sex	Female	2309 (33.4%)
	Age	Mean ± SD (years)	58.1 ± 21.7
	Trauma mechanism	TA car	599 (8.7%)
		TA motorcycle	266 (3.9%)
		TA bicycle	809 (11.7%)
		TA pedestrian	398 (5.8%)
		Fall > 3 m	759 (11.0%)
		Fall < 3 m	2984 (43.2%)
		Other	918 (13.3%)
	Trauma type	Blunt	6378 (92.4%)
	ISS	Mean ± SD	20.7 ± 9.1
	TBI characteristics	Epidural hematoma	708 (10.3%)
		Subdural hematoma	3475 (50.3%)
		Subarachnoid hemorrhage	2424 (35.1%)
		Intracerebral hemorrhage	4077 (59.0%)
		Edema/swelling	1118 (16.2%)
		Brainstem hemorrhage	392 (5.7%)
		Skull fractures	3602 (52.1%)
	AIS head	3	1973 (28.6%)
		4	2006 (29.1%)
		5	2840 (41.1%)
		6	85 (1.2%)
	GCS	Median (IQR)	7 (3–11)
	Motor response	Normal	665 (9.6%)
		Specific	2980 (43.2%)
		Nonspecific	931 (13.5%)
		None	2328 (33.7%)
	Pupil reactivity	Brisk	3343 (48.4%)
		Sluggish	2058 (29.8%)
		Fixed	1506 (21.8%)
	Pupil size	Normal	4640 (67.2%)
		Anisocoric	1331 (19.3%)
		Bilaterally dilated	933 (13.5%)
	Reanimation	Present	94 (1.4%)
	MSCT	Present	5056 (73.2%)
Timing	Time from admission to first CT	Mean ± SD (min)	19.0 ± 12.2
	- Level 1 trauma center	Mean ± SD (min)	18.7 ± 11.8
	- Level 2 trauma center	Mean ± SD (min)	21.5 ± 14.7
	Time from trauma to hospital	Mean ± SD (min)	68.8 ± 31.1
	Total time in trauma room	Mean ± SD (min)	52.0 ± 31.6
Outcome	Predicted mortality (RISCII)	Mean ± SD (%)	29.3 ± 32.2
	Mortality	Total	2187 (31.7%)
		within 24h	1005 (14.6%)
		within 6h	347 (5.0%)
	Length of stay in hospital	Mean ± SD (days)	15.0 ± 15.0

*AIS* Abbreviated Injury Scale, *CT* computed tomography, *GCS* Glasgow Coma Scale; *ISS* Injury Severity Score, *IQR* interquartile range, *MSCT* multislice computed tomography indicating whole-body CT, *RISC-II* Revised Injury Severity Classification II, *SD* standard deviation, *TA* traffic accident

**Fig. 2:**
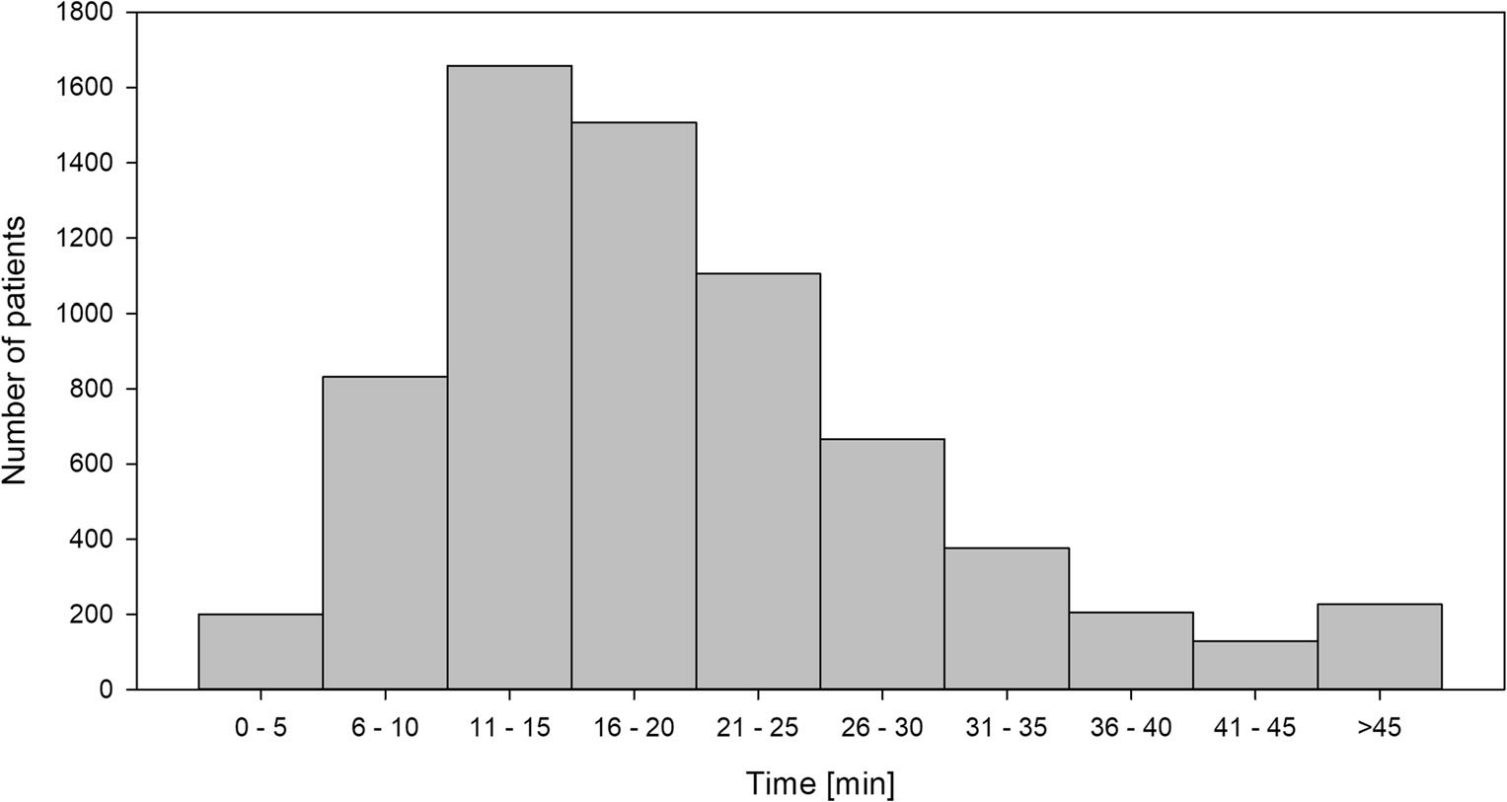
Distribution of times from admission of the patient until the CT scan. CT computed tomography

**Fig. 3:**
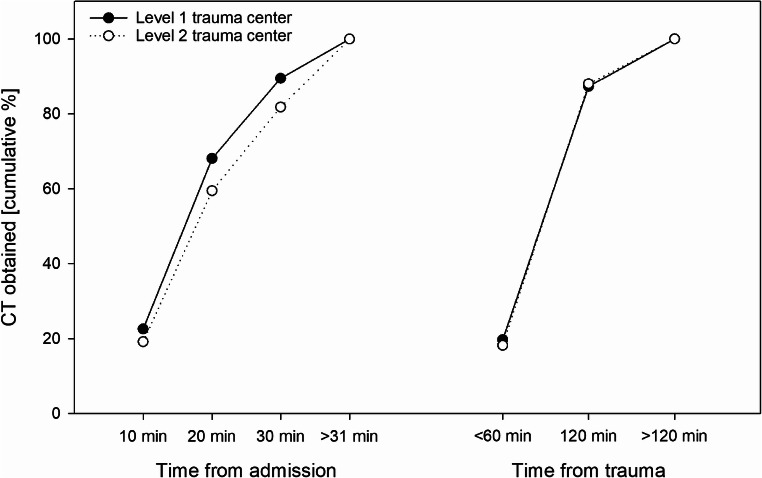
Line plot of times from admission and from trauma to CT in level 1 and 2 trauma centers. CT computed tomography

Table 2 depicts subgroups of the study cohort dependent on different timeframes from admission to CT. Most cases demonstrated a time period of 11 to 20 min (44.9%) whereas a period of more than 30 min was present in only 11.3% of cases (Fig. 2). Trauma appeared slightly more severe in groups with shorter timeframes: mean ISS was 21.3 and 20.2, median GCS was 6 and 8 and mean age of years was 56.8 and 60.1 in the ≤ 10-min and ≥ 31-min groups, respectively. Patients of the ≤ 10-min group received the lowest rate of additional imaging like abdominal sonography (64.2% vs. 81.6% overall) or chest X-ray (21.0% vs. 40.5% overall). Mortality was the highest in the ≤ 10-min group (32.4%) and the lowest in the ≥ 31-min group (30.0%). The distribution of pupil status changed steadily over the length of the timeframe from 63.2%, 21.2%, and 15.7% in the ≤ 10-min group to 76.6%, 13.9%, and 9.5% in the ≥ 31-min group for normal, anisocoric, and bilaterally dilated pupils, respectively. Overall, the time from the admission to the execution of the CT examination in level 1 centers was faster than in level 2 centers (Fig. 3).

**Table 2:** Demographic, clinical, and outcome data of patients distributed of time from admission of the patient to first CT in minutes. Time of trauma to hospital was available for 5259 patients (76.1%)

		Time from admission to first CT (min)				
		≤ 10	11–20	21–30	≥ 31	Total
Total		1539 (22.3%)	3106 (45.0%)	1483 (21.5%)	779 (11.3%)	6907
Age	Mean ± SD (years)	56.8 ± 21.7	58.1 ± 21.7	58.6 ± 21.6	60.1 ± 21.5	58.1 ± 21.7
ISS	Mean ± SD	21.3 ±9.7	20.6 ± 9.1	20.5 ± 9.0	20.2 ± 8.5	20.7 ± 9.1
AIS head	5 and 6	696 (45.2%)	1303 (42.0%)	631 (42.5%)	296 (38.0%)	2924 (42.4%)
GCS	≤ 8	973 (64.4%)	1891 (62.0%)	852 (59.1%)	390 (52.8%)	4102 (60.9%)
Pupils bilaterally dilated	Present	241 (15.7%)	430 (13.9%)	188 (12.7%)	74 (9.5%)	933 (13.5%)
Systolic blood pressure	Mean ± SD (mmHg)	138.0 ± 38.7	138.7 ± 39.3	137.6 ± 39.9	137.4 ± 38.2	138.2 ± 39.2
Abdominal sonography	Present	987 (64.2%)	2696 (86.8%)	1311 (88.4%)	643 (82.5%)	5632 (81.6%)
Chest X-ray	Present	323 (21.0%)	1237 (39.8%)	795 (53.6%)	443 (56.9%)	2794 (40.5%)
Reanimation	Present	19 (1.3%)	39 (1.3%)	21 (1.4%)	15 (2.0%)	94 (1.4%)
Time: trauma to hospital	Mean ± SD (min)	70.8 ± 32.3	69.1 ± 31.3	67.7 ± 29.2	65.5 ± 30.8	68.8 ± 31.1
Time: admission to first CT	Mean ± SD (min)	7.5 ± 2.5	15.5 ± 2.9	25.0 ± 2.8	43.7 ± 16.3	19.0 ± 12.2
Mortality	Present	497 (32.3%)	1001 (32.2%)	455 (30.7%)	234 (30.0%)	2186 (31.7%)
Length of stay in hospital	Mean ± SD (days)	14.9 ± 13.8	14.7 ± 15.5	15.0 ± 14.2	16.3 ± 16.4	15.0 ± 15.0

*AIS* Abbreviated Injury Scale, *CT* computed tomography, *GCS* Glasgow Coma Scale, *IQR* interquartile range, *ISS* Injury Severity Score, *SD* standard deviation

**Fig. 4:**
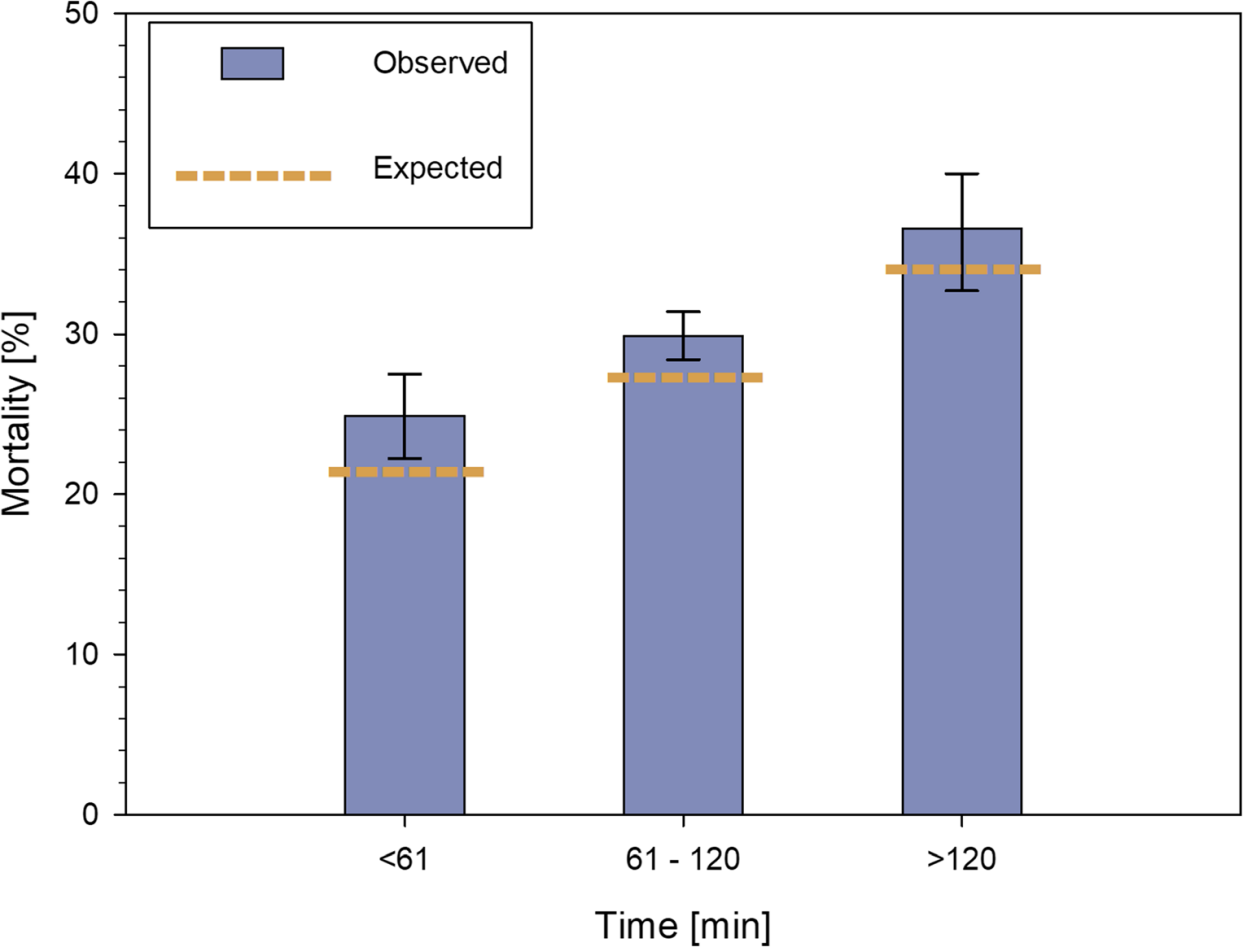
Mortality (bar with 95% confidence interval) and RISC-II prognosis (horizontal line) distributed on the time until the CT examination. RISC-II Revised Injury Severity Classification II

In 5259 of 6907 patient (76.1%) data were available for timeframe from trauma to initial CT. The average duration from trauma to initial CT was 87.4 ± 33 min with a duration between 61 and 120 min for 67.8% of the patients. A CT scan within an hour from trauma was performed in 19.5% of patients whereas a time period of more than 2 h was present in 12.7% of cases. Table 3 depicts the characteristics of these subgroups. Trauma severity and age were higher in groups with a longer timeframe. Mean ISS was 19.8 ± 9.0, 20.7 ± 9.3, and 21.4 ± 7.5 in the ≤ 60-min, 61–120-min, and ≥ 121-min groups, respectively. Equally, median GCS was the highest in the ≤ 60-min group (9 IQR 4-12) and the lowest in the ≥ 121-min group (6 IQR 3-10). Mortality exhibited a similar distribution of 24.9%, 29.9%, and 36.3% in the ≤ 60-min, 61–120-min, and ≥ 121-min groups, respectively, and is also depicted in Fig. 4. In contrast to the faster times between admission of the patient and performing the CT between the levels of the trauma centers, there was no relevant difference between the times from accident to CT (Fig. 3).

**Table 3:** Demographic, clinical, and outcome data of patients distributed of time from trauma of the patient to first CT in minutes. Time of trauma to hospital was available for 5259 patients (76.1%)

		Time from trauma to first CT (min)			
		≤ 60	61–120	≥ 121	Total
Total		1027 (19.5%)	3566 (67.8%)	666 (12.7%)	5259
Age	Mean ± SD (years)	54.8 ± 21.3	57.0 ± 21.9	61.2 ± 20.9	57.1 ± 21.8
ISS	Mean ± SD	19.8 ± 9.0	20.7 ± 9.3	21.4 ± 7.5	20.6 ± 9.0
AIS head	5 and 6	356 (34.7%)	1443 (40.5%)	329 (49.4%)	2128 (40.5%)
GCS	≤ 8	493 (49.5%)	2135 (61.1%)	438 (66.8%)	3066 (59.6%)
Systolic blood pressure	Mean ± SD (mmHg)	137.8 ± 36.1	136.8 ± 38.9	139.3 ± 42.0	137.3 ± 38.8
Abdominal sonography	Present	798 (77.8%)	2930 (82.2%)	548 (82.3%)	4276 (81.4%)
Chest X-ray	Present	354 (34.5%)	1484 (41.6%)	288 (43.2%)	2126 (40.4%)
Reanimation	Present	12 (1.2%)	46 (1.3%)	11 (1.7%)	69 (1.3%)
Time: trauma to hospital	Mean ± SD (min)	37.2 ± 9.2	67.4 ± 17.3	125.0 ± 34.8	68.8 ± 31.1
Time: admission to first CT	Mean ± SD (min)	13.2 ± 6.4	18.8 ± 10.2	26.1 ± 20.6	18.7 ± 12.0
Mortality	Present	255 (24.9%)	1067 (29.9%)	242 (36.3%)	1564 (29.8%)
Length of stay in hospital	Mean ± SD (days)	14.3 ± 11.9	15.2 ± 15.3	15.7 ± 15.0	15.1 ± 14.8

*AIS* Abbreviated Injury Scale, *CT* computed tomography, *GCS* Glasgow Coma Scale; *ISS* Injury Severity Score, *SD* standard deviation

To explore the influence of CT timing on mortality, SMRs were calculated (Table 4 and Fig. 4). Generally, the observed mortality of the overall cohort (31.7%) was higher than the predicted mortality according to RISC-II (29.3%, Table 1). With regards to time period from admission to CT, a mortality ratio higher than expected was present in the 11–20-min (1.11, 95%CI 1.05–1.17) and the ≥ 31-min group (1.13, 95%CI 1.00–1.25). The groups of ≤ 60 min and 61–120 min from trauma to CT also showed elevated mortality ratios of 1.14 (95%CI 1.01–1.26) and 1.08 (95%CI 1.02–1.13), respectively.

**Table 4:** Observed versus predicted mortality using the RISC II and standardized mortality ratio

		Observed mortality (%)	Predicted mortality based on RISC II (%)	SMR (95%CI)
Time from admission to first CT (min), *n* = 6907	≤ 10	32.4	31.2	1.04 (0.96–1.11)
	11–20	32.2	29.1	1.11 (1.05–1.17)
	21–30	30.7	29.1	1.05 (0.97–1.14)
	≥ 31	30.0	26.7	1.13 (1.00–1.25)
Time from trauma to first CT (min), *n* = 5259	≤ 60	24.9	21.9	1.14 (1.01–1.26)
	61–120	29.9	27.8	1.08 (1.02–1.13)
	≥ 121	36.3	34.5	1.05 (0.95–1.16)

*CT* computed tomography, *CI* confidence interval, *RISC-II* Revised Injury Severity Classification II, *SMR* standardized mortality ratio

Finally, the multivariable logistic regression analysis demonstrated that treatment at a level 2 trauma center was associated with slightly but statistically significant increased mortality risk (odds ratio (OR) 1.280; CI 1.015–1.614). Again, time from admission to CT had no significant impact in this model (OR 1.002; CI 0.996–1.008) (Table 5).

**Table 5:** Multivariate regression analysis for Level 2 trauma center treatment and time to CT from admission adjusted for the RISC

Mortality	Coefficient	*p* value	OR	95%CI
RISC-II score	− 0.973	< 0.001	0.378	0.361–0.396
Level 2 trauma center	0.247	0.037	1.280	1.015–1.614
Time to CT from admission (min)	0.002	0.528	1.002	0.996–1.008
Constant	0.143	< 0.001	0.30	

*CT* computed tomography, *CI* confidence interval, *OR* odds ratio, *RISC-I*, Revised Injury Severity Classification II

## Discussion

The need for a rapid CT scan to determine a traumatic brain injury as part of the trauma management is obvious. A 24/7 availability of a CT scan with radiological assessment is required. This should be performed within 20–30 min after admission of the patient in order to be able to provide early surgical therapy in case of intracranial hematoma to lower intracranial pressure and minimize secondary brain damages [2, 10, 17].

Surprisingly, very little data has been published on this topic so far, making this study driven from the TR-DGU one of the largest investigations of the impact of the duration from admission to first computer tomography scan in moderate-to-severe traumatic brain injury.

Timeframes from trauma and from admission to the CT scan were 87.4 and 19.0 min in our study cohort, respectively, which appears short in comparison with other studies [2, 4, 12, 15, 16, 19].

Overall, however, the comparability of the results is only possible to a very limited extent, since the studies differ in the composition of the cohort, which is apparent from different ISS. Moreover, some studies showed marked variation in the rate of abdominal ultrasound examinations and chest X-ray examinations. Accordingly, with an increased rate of additional diagnostic measures, the average time taken to perform the CT scan was longer [9, 18]. This fact is also evident in our study, as time to CT increased with an increase in the rate of additional examinations.

Another factor influencing the time between admission and first CT scan could be the proximity of the CT to the trauma room. However, results of studies addressing this issue are divergent. The REACT-1 study demonstrated that a CT scanner located in the trauma room reduced the duration (24 vs. 38 min) until the first imaging but was not associated with statistically significant beneficial outcome in severe TBI patients [18]. In contrast, Huber-Wagner et al. observed that the location of a CT scanner in the trauma room was associated with a mean reduction of 5 min (17.1 vs. 22.7 min) compared to a CT equal or less than 50 m away and was associated with a reduction of the observed mortality [9]. Surprisingly, not only the associated outcome but also the timing of the CT scan itself is reported heterogeneously in further studies evaluating the positioning of a CT scanner in the trauma room [7, 13]. In a prospective single-center study, time spans were compared before and after installation of a new CT scanner demonstrating a minimal prolongation in the new setting (20 vs. 21 min) [20]. In contrast, the installation of a hybrid emergency room at another institution was associated with shorter times to the first imaging (25 vs. 14 min), earlier initiation of emergency surgery, and improved functional outcome in patients with severe TBI [11]. These different results suggest that in addition to the spatial distance of the CT scanner from the trauma room, local treatment algorithms seem to determine the time period before imaging [9]. As a consequence, the TraumaNetzwerk DGU® (trauma networks; TNW) and guidelines were introduced to standardize the treatment algorithms and to optimize the treatment level for trauma patients in Germany. High standards are requested from participating hospitals regarding personal, organization, and infrastructure. For example, immediate proximity of the trauma room to the radiology department is mandatory for level I trauma centers and proximity is recommended for level II centers [3]. The guideline also includes recommendations when to perform abdominal sonography or chest X-ray examinations in the trauma room. Depending on the suspected severity of trauma, this might result in a higher frequency of these diagnostic measures which is naturally also reflected in an extended time period until a CT scan is performed. However, we show that longer time spans do not lead to a significant increase of the SMR. This may be interpreted as support for implemented guidelines since delays in acquiring cerebral imaging seem to be justified and to serve the adequate management of the trauma patient. Another notable finding of the study is that emergency room staff obviously pays attention to the admission status of the pupils as the rate of patients with anisocoric or bilaterally fixed and dilated pupils was associated with faster acquisitions of the CT. Overall, adherence to guidelines and recommendations can demonstrably lead to an improvement in care in patients with TBI and offer the opportunity for quality improvements, especially in process management [2, 5, 6, 10].

The strength of the study is, on the one hand, the relatively high number of cases and, on the other hand, the multicenter approach through the use of data from the TR-DGU which allows a uniform and standardized collection of time data. Moreover, compared to other studies, the inclusion and exclusion criteria available from the TR-DGU allowed for a relatively homogeneous patient population to be evaluated.

This fact leads directly to the limitations of the study, namely, that an analysis of individual hospital data sets is not meaningfully possible and possible differences between the local treatment algorithms could have been shown to have a statistically significant effect on mortality. Furthermore, the study has the well-known limitations of a retrospective analysis; despite the fact that the data sets are prospectively entered into the TR-DGU, the results demonstrate associations and no causalities as mentioned before [8, 9]. A usual issue of large-scale databases is the risk of incomplete or incorrect data despite various electronic plausibility checks when entering online data, which may have affected the presented results. However, it should be emphasized that the data entry for the times of admission of the patient as well as the CT examination are clearly defined and standardized for the TR-DGU. Another limitation of the TR-DGU is the lack of detail with regard to the pathology of the TBI, so that no detailed statements, e.g., the extent of hemorrhages, are available and only a categorization based on the AIS can be made which is needed for the adjustment in the RISC-II score. Finally, the RISC-II score has been validated for mortality prediction of a large number of patients. However, it is not validated for special subgroup analyses like TBI patients [14].

## Conclusion

TBI patients exhibiting a longer time span from trauma to first CT were more severely injured and demonstrated a worse prognosis. Conversely, patients with more severe injuries received a CT scan faster once admitted to the hospital. Surprisingly, the time span from admission to the CT scan itself showed no significant impact on the mortality in this cohort of the TR-DGU. It might be concluded that time management of trauma patients with TBI as leading injury in out-of-hospital and early clinical setting is adequate leading to timely initial diagnostic measures without causing harmful delay.

## Data Availability

All epidemiological data presented in this study were retrieved from the TraumaRegister DGU® of the German Trauma Society. The data that support the findings of this study are available from TraumaRegister DGU® but restrictions apply to the availability of these data, which were used under license for the current study, and so are not publicly available.
